# Radiation therapy for glomus tumors of the temporal bone

**DOI:** 10.1016/S1808-8694(15)31244-1

**Published:** 2015-10-20

**Authors:** Celso Dall'Igna, Marcelo B. Antunes, Daniela Pernigotti Dall'Igna

**Affiliations:** Joint Professor of Otorhinolaryngology, UFRGS - Ph.D. In Medical Sciences, Head of the ENT Services at Clinical Hospital in Porto Alegre; Otorhinolaryngology-Physician, Fellow of Fisch Foundation; Internist, Otorhinolaryngologist, Federal University of Paraná. Service of Otorhinolaryngology and Head and Neck Surgery – Hospital das Clínicas de Porto Alegre - Medical School, Federal Universidade of Rio Grande do Sul

**Keywords:** radiotherapy, ear, hearing loss, paraganglioma, quemodectoma

## Abstract

The treatment of glomic tumors has been controversial since its first description. It can be done with surgery, radiotherapy or just expectation.

**Aim:**

The objective of this paper was to evaluate the effectiveness and complications of radiotherapy.

**Study design:**

clinical with transversal cohort.

**Material and Method:**

It was made a retrospective review in the charts of the patients with glomus jugulare tumors treated with radiotherapy. Disease control was determined by (1) no progression of symptoms or cranial nerve dysfunction or (2) no progression of the lesion in radiological follow-up. It was also evaluated the follow-up period and the sequelae of the treatment.

**Results:**

Twelve patients were included, 8 of then women. The follow-up period was from 3 to 35 years, with a media of 11,6 years. The main symptoms were: hearing loss, pulsate tinnitus, dizziness and vertigo. The signs were pulsate retrotympanic mass, facial palsy and cofosis. The tumors were staged using Fisch's classification. The radiotherapy was performed with linear accelerator with dose ranging from 4500-5500 in 4–6 weeks. In the follow-up period were possible to identify sequelaes like dermatitis, meatal stenosis, cofosis and facial palsy.

**Discussion:**

The signs and symptoms were the same found in the medical literature. The type and dosages of the radiotherapy were also the same of others reports. All patients had improvement of the symptoms and only one was not considered as having disease controlled. Complications were, in general, minor complications, with exception of the cofosis and facial palsy.

**Conclusion:**

Radiotherapy is a viable alternative to treatment of these tumors because their good response and low level of complications. It should be considered specially in advanced tumors where a surgical procedure could bring a high level of morbidity.

## INTRODUCTION

Glomus tumors also known as paragangliomas or chemodectomas are the most common neurological neoplasm after acoustic neuroma. These tumors are originated from glomus bodies or paraganglia, which are structures from the neuroendocrine extra-adrenal system usually related to sympathetic ganglia.

The treatment of these tumors has been subject of controversy since the first study published in the 40's by Guild^1^ & Rosenwasser^2^, and may be divided into curative and palliative. The only curative treatment available is surgery, and the palliative treatment may include radiotherapy or wait and watch protocol. Generally these tumors are addressed on individual basis according to the age of the patient, the size, type and staging of the tumor.

The objective of this study was to evaluate radiation effectiveness and radiotherapy complications for glomus tumor of the temporal bone.

## MATERIAL AND METHOD

This study included 12 patients with glomus jugulare tumor of the temporal bone treated with radiotherapy between 1960 and 2001 that have been periodically followed up at the Otorhinolaryngology Service of Hospital das Clínicas de Porto Alegre. The last appointment occurred between January 2004 and February 2005. The patients with glomus tympanic tumor and those that have exclusively undergone surgery were excluded from the study.

Patients referred to radiotherapy were those that refuse to undergo surgical treatment, those with lesions considered non-resectable or those that did not have clinical conditions to undergo general anesthesia. Control criteria of the diseased through radiotherapy were (1) absence of progression of symptoms or cranial nerve dysfunction, and (2) the lesion did not increase according to physical examination or radiological control.

The analysis of the charts of the patients included information related to personal data (age and gender), clinical history, physical examination and audiometry tests on diagnosis, initial staging, proposed and conducted treatment, and follow-up of the patients. Radiological findings were recovered through expert's opinion made by the radiologist. The staging of the tumor was classified according to Fisch^3^ (Table 1). Radiation therapy concerning the type, dose and treatment interval was analyzed based on final opinion of the radiotherapist after the end of the treatment. The follow up of the patients was conducted on a consistent basis: monthly reviews during the first 6 months, then quarterly reviews up to complete 2 years, six months review until 5 years of treatment, and annual reviews after it. At every appointment patients answered questions about symptoms (hypoacusia, pulsatile tinnitus, dizziness/vertigo, otorrhea, otalgia, post-radiotherapy symptoms and others) it included a detailed and careful description of the otoscopy findings. All patients have undergone CT (Computerized Tomography) annually to evaluate any likely progression of the tumor.

**Table 1:** Classification of glomus jugularis tumors of the temporal bone as proposed by Fisch (4).

Type	Description
A	Tumors restricted to middle ear (glomus tympanicum tumors)
B	Tumors restricted to tympanomastoid site
C	Tumors involving the infra-labyrinth portion towards the petrous apex
D1	Tumor with intracranial invasion (< 2 cm)
D2	Tumor with intracranial invasion (> 2 cm)

## RESULTS

This study carried out in 12 patients included 8 women (mean age at diagnosis was 50) aged from^31–87^ years. The follow up interval ranged from 3 to 35 years (mean of 11.6 years). Major symptoms and findings in physical examination at diagnosis are included in Table 2. The audiometric findings were anacusia in two thirds of the patients, mixed loss in 2 patients and conductive loss in 1 patient. The audiometry of one patient at diagnosis was not recovered, and the oldest one available was that performed after radiotherapy showing mixed hearing loss in the ear affected by the disease. The patient mentioned there was no significant change in hearing after treatment.

**Table 2:** Signs and symptoms on diagnosis.

Symptoms	N	%
Hypoacusia	12	100
Pulsatile Tinnitus	12	100
Dizziness/vertigo	8	66,6
Otorrhea	5	41,6
Otalgia	1	8,3
Facial Palsy	5	41,6
Pulsatile retrotympanic mass	8	66,6
Polypoid mass of the		
External Auditory canal (CAE)*	4	33,3

* CAE = external auditory canal

Data related to each patient are summarized in Table 3.

**Table 3:** Patients that received radiotherapy to treat glomus tumors.

‘Name						
Age	Symptoms	Physical Examination	Computerized Tomography	Est	Rxt	Follow-up
Gender						
IGA 70 F	Pulsatile Tinnitus, Dizziness & Left Ear hypoacusia. Slow progression of Symptoms Over the last Two years	Red Mass in Inferior quadrants of MT Cofosis on the Left Ear.	Expansive lesion of the jugular foramen To left with bone mastoid destruction Hypotympanum, labyrinth and External Auditory Canal. Erosion of the inferior portion of the Internal Auditory Canal and Skull base	C	AL	(12/2004) Tinnitus Pulsatile and mild Hypoacusia. Reddish Non-pulsatile mass CT with process Stabilization
NRB 61 F	Pulsatile tinnitus, Progressive Hypoacusia of Right Ear Dizziness. Facial palsy for 1 year. Otorrhea.	Pulsatile polyp in External Auditory Canal. Facial Palsy level III Cofosis.	Expansive lesion of Right jugular foramen with bone erosion Of External Auditory Canal, Mastoid, labyrinth, Facial canal and adjacent carotid canal	C	AL	(02/2005) Mild pulsatile Tinnitus and Hypoacusia. Reddish pulsatile mass. CT with process Stabilization. Otalgia.
LCR 87 F	Pulsatile tinnitus & Hypoacusia on Left Ear.	Pulsatile polyp in External Auditory Canal. Facial palsy grade III. Cofosis.	Expansive lesion of Left Jugular foramen extending to mastoid, hypo and mesotympanum, labyrinth & petrous apex with no invasion Of Internal Auditory Canal CAI.	C	AL	(04/2004) Mild non-pulsatile Tinnitus Hypoacusia and vertigo. Mass found in otoscopy Pulsatile. CT stable.
DMA 43 M	Left Ear Pulsatile tinnitus Hypoacusia. Dizziness with Vertigo seizures. Facial palsy.	Pulsatile Mass Occluding External Auditory Canal LE	Expansive lesion Left Jugular foramen with mastoid bone destruction, Hypotympanum, labyrinth and External Auditory Canal Erosion of the inferior portion of the Internal Auditory Canal and skull base	D1	AL	(08/2004) Sustained Hypoacusia Improvement of Dizziness and tinnitus. CT With no progression signs External Auditory Canal Stenosis
NMZ 73 F	RE Pulsatile tinnitus & hypoacusia Dizziness. Facial Palsy (03 years).	Massreddish behind TM. Facial Palsy (level III) Mixed loss RE	Expansive lesion jugular foramen to the Right extending to Mesial and Hypotympanum Opacifying the mastoid.	B	AL	(01/2004) Expansive lesion jugular foramen to the Right extending to mesial and Hypotympanum Opacifying the mastoid.
CSM 43 F	Pulsatile tinnitus, hypoacusia and RE otalgia. Occasional Dizziness Worsening over the last 2 years	Pulsatile reddish Mass Behind TM Conductive Loss 20dB.	Expansive lesion jugular foramen to the Right extending to mesial and hypotympanum Eroding mastoid and carotid of adjacent canal	C	TC o	(01/2005) Pulsatile Tinnitus Bloody otorrhea Pulsatile Mass Behind TM Desquamation of External Auditory Canal Cofosis.
DS 46 M	Pulsatile tinnitus and hypoacusia RE Dizziness & Unbalance. Occasional Otorrhea.	Pulsatile Polyp in CAE (RE) Cofosis.	Expansive lesion of Right jugular foramen with destruction of Mastoid structures of the internal ear Extended to Infratemporal fossa and Right Cerebellar Hemisphere	D2	AL	(11/2004) Mild non pulsatile Tinnitus and Stable hypoacusia CT With no signs of progression Facial Palsy External Auditory Canal Stenosis pruritus.
EC 67 M	Pulsatile tinnitus, hypoacusia and Dizziness. Occasional Otorrhea	Pulsatile reddish Mass Behind TM RE. Cofosis.	Expansive lesion Involving mastoid & cochlea, adjacent to carotid Canal.	B	AL	(03/2004) Non pulsatile Tinnitus Stable Hypoacusia Reddish Mass Behind TM Non-pulsatile.
JMG 67 M	Palatal tinnitus, hypoacusia, otorrhea Bloody. Facial palsy	Reddish Pulsatile Mass in LE CAE Facial palsy (level II) Cofosis.	Expansive lesion Left jugular foramen extending to mesotympanum and CAE with Bone erosion of the petrous region and IAC Fossa Invasion posteriorly Sparing meninges	D1	AL	(11/2004) Mild pulsatile Tinnitus and Stable Hypoacusia Reddish Mass behind MT. Dermatitis after radiotherapy
NL 60 F	LE Hypoacusia, bloody otorrhea	Reddish Pulsatile Mass Bulging TM Facial Palsy. Cofosis.	Expansive lesion of Left jugular foramen with invasion of middle ear and CAE Internal ear preserved Involve internal Carotid.	B	AL	(12/2004) Mild Tinnitus, hypoacusia Stable CT with no Progression of lesion Nor CAE stenosis.
NSO 75 F	LE Pulsatile tinnitus hypoacusia Non-rotation Dizziness	Reddish Pulsatile Mass Bulging TM Cofosis LE	Expansive lesion next to Left jugular gulf Extending to-carotid canal, mastoid and tympanic cavity With destruction of basal cochlear spiral	C	AL	(11/2004) Stable Hypoacusia And mild pulsatile tinnitus Pulsatile Mass In inferior quadrants Progression-free CT Stable lesion Dermatitis
LLD 61 F	RE Pulsatile tinnitus and Hypoacusia. Facial palsy.	Reddish Pulsatile Mass Bulging TM Facial palsy level V. Cofosis.	Expansive lesion next to Left jugular gulf Extending to carotid canal, mastoid Tympanic cavity with cochlear and petrous portion erosion Posterior fossa Invasion.	D1	AL	(01/2004) Tinnitus (1/2004) Stable Hypoacusia And mild Pulsatile tinnitus Pulsatile Mass In inferior quadrants Stable CT Lesion, dermatitis

Est: staging, Rxt: Radiotherapy, AL: Linear accelerator, TCo: telecobalt, CAE: External Auditory Canal, TM: Tympanic Membrane.

Radiological findings were extremely variable due to multidirectional growth of these tumors. The analyses of such exams provide us with the opportunity to evaluate tumor extension and perform an accurate staging of such lesions. Tumor staging was performed according to Fisch's classification in Table 4.

**Table 4:** Patients' Staging

Staging	N (%)
A	0
B	3 (25%)
C	5 (41,6%)
D1	3 (25%)
D2	1 (8,3%)

The type radiation therapy used was megavolt radiation with linear accelerator and dose ranging from 4,500 to 5,500 Rads during 4 to 6 weeks. Only one patient (CMS) received therapy with telecobalt for 8 weeks and dose of 5,200 Rads.

The last appointment follow-up dates were between January 2004 and February 2005 when data collection was finished. Most of the patients mentioned symptoms only if they were asked about any specific complaints during protocol application. The hearing loss remained stable before and after radiation therapy in those patients with mixed loss (2 patients); however deafness after radiation therapy occurred in one case of a patient with conductive loss. In general, tinnitus was described and did not cause any discomfort for patients, it remained pulsatile in 6 patients even after radiotherapy. Physical examination showed the presence of retrotympanic tumor in most of the patients, and in 41.6% of them the mass was pulsatile.

Radiological controls did not show any progression of the disease in all patients, except for one (CMS). This patient had undergone CT scan in Dec/2004 showing mild increase in the lesion with density of soft parts that occupied the tympanic cavity and part of the External Auditory Canal. Since the patient did not have any new complaints and does not want to undergo rescue surgery, he/she is under clinical follow-up. During the follow-up period of patients it was possible to find out that all patients presented, at different and variable levels, one or more effects resulting from radiotherapy. The most common one was dermatitis with severe desquamation and fragility of the External Auditory Canal skin that occurred in half of the patients. The stenosis of the External Auditory canal was reported in 25% of the patients during follow-up. Other complications such as otalgia, vertigo, cofosis and facial palsy occurred only once. One patient eventually presented a basal cell carcinoma in the face and neck approximately 30 years after radiotherapy on the same irradiated side.

## DISCUSSION

Surgery is advocated by many as the ideal treatment^4^^,^^8^. It is justified due to technical developments of skill base surgery, tumor resectability has enabled good functional results, decreased effects and good quality of life postoperatively. Recurrence indexes varied from 5.5 to 54%.^4^^,^^17^^,^^18^ Surgery morbidity is high and fairly variable, leading to primarily cranial nerve dysfunction and liquor fistula. In one study of Jackson et al. 4, lesion index of cranial nerves just after the surgery reached 59% of patients. It was reported that total removal of the tumor without affecting cranial nerves was referred as possible in only 31% of the cases. Cace et al. 19 had lesion at least of some cranial nerve in all patients. Hawthorne et al 20 reported 47% of cranial nerve dysfunction preoperatively and 95% dysfunction postoperatively. Liquor fistula was found in 12 to 64% of the patients^4^^,^^19^^,^^20^. Gardner et al. 8 reported 50% of dysphagia, 19% of facial palsy postoperatively and 19% of liquor fistula. Perioperative mortality varied from 2.7 to 3%^4^^,^^21^. Surgery resulted in cure of the disease in 54 to 85% of the cases^4^^,^^9^^,^^16^, however, long-term effectiveness still needs to be determined.

Others view radiotherapy as the initial treatment^5^^,^^9^^,^^11^ due to the morbidity of surgical procedure. The objective is to have local/regional control of the disease in the long term, without any subjective our objective progression of the disease. Typically disease control is defined as in the current study: absence of progression of symptoms or cranial nerve dysfunction without any lesion increase upon physical examination or radiological control. Tumors may present a decrease in size, but rarely disappear as observed in pathophysiological analysis of follow-up and radiological tests. A study from Spector et al.^6^ showed that chief cells are not radiosensitive, remaining viable after radiotherapy. These cells, however, no longer presented mitotic activity. The stroma showed itself as more reactive to radiotherapy and was replaced by fibrous tissue rich in fibroblasts, similarly to vascularization in which one may observe changes such as perivascular fibrosis, endothelial hyperplasia and subendothelial degeneration.

In the presented series it was possible to find 2:1 ratio favorable to women, which is somewhat different from the literature that sets a ratio of 4 to 7:1^4^^,^^9^. The age range of onset of tumors was 43 to 87 years (mean 62.4 years) a little higher then reported in literature, with higher incidence of tumors occurring between 5th and 6th decades. Mean follow up considered as appropriate was more than 11 years to determine a definitive response to the treatment, although it is known that glomus tumors have slow growth rate and some reports mention a recurrence of tumors even after twenty years^9^.

Symptoms and findings of physical and additional examinations were not different from those reported in the literature^14^^,^^15^, with a long interval between the onset of symptoms and diagnosis^14^. None of the patients obtained improvement in symptoms such as hearing loss, tinnitus or cranial nerve dysfunction after radiotherapy, as found in other series^13^^,^^22^.

The type of radiotherapy applied was linear accelerators. The dose and term of the radiotherapy were also not different from those used in routine medical practice^7^^,^^9^^,^^12^^,^^13^. Some studies used irradiation with cobalt, whereas others used linear accelerator. Both radiotherapy types are considered as megavoltage radiotherapy, and a difference in results was not expected between one and the other.

Most of the studies about radiotherapy for glomus tumors did not explicitly refer complications, only the term “few complications” was mentioned. In the investigated series it was possible to observe that all patients had some degree of complications. Actinic dermatitis after radiotherapy may really be considered a minor event, and may be controlled with skin medicines and frequent cleaning of the External Auditory Canal. On the other hand, the presence of stenosis in soft tissue of the External Auditory Canal cannot be neglected since it may hinder proper postoperative examination, and affect hearing as well (although most of the patients had anacusia in the affected ear).

Cofosis and facial palsy after radiotherapy are considered major complications of radiotherapy. The patient with hearing loss symptom after treatment (CMS) was irradiated with telecobalt (5,200 Rads) for 26 days; this dose is not considered high if compared against data from the literature. The same patient was the only one considered as therapeutic failure since there was increase in the tumor in the follow-up tomography.

In other series, many patients were treated with cobalt at similar doses and reported good results without any significant complications. Therefore, the treatment should not be held responsible for complications or treatment failure. The patient developed facial palsy and had a tumor with extensive bone destruction and intracranial involvement, and palsy could have resulted from the invasion of the nerve since only tumor abnormalities could be found after radiotherapy. In the patient that had basal cell carcinoma it was not possible to determine if it was a consequence of radiotherapy applications or if it would develop anyway since the patient is a rural worker and had long history of sunlight exposure.

The control of the disease in our series was considered satisfactory and successful in 91.6% of the cases. Other authors also demonstrated long-term control of glomus jugulare tumor with radiotherapy. They report success rates ranging from 84 to 98% with the recommended dose. Hatfield et al 23 had 100% control in 16 patients with doses ranging from 4,000 or above cGY and also reported that each 1 out of 2 respected cases that had cure as the objective, relapsed. Kim^12^ demonstrated that radiotherapy alone had an 88% rate of disease control, whereas post-resection subtotal radiotherapy had 85% of disease control. Larner presented a study carried out in 49 patients. Twenty had undergone surgery, 14 had undergone surgery and postoperative radiotherapy, and 15 were treated only with radiotherapy, and the other surgeries associated or not with post-operative radiotherapy. Disease control with radiotherapy was 93%. Radiotherapy has not received the recognition it deserves because new techniques that use megavoltage are not widely disseminated in clinical practice yet. In the past, orthovoltage was highly related with frequent complications and lack of predictable response in disease control. Another key aspect was the fact that “chief cells” are radio resistant, and it brings some level of concern, but at the same time one should bear in mind that the vascular portion of the tumor presents fibrosis only after irradiation. The lack of a consistent definition about the success in treating glomus jugulare tumors in the literature affects the comparisons between different treatment regimes.

## CONCLUSION

As to radiation treatment for glomus jugulare tumors, since it presents a good response in disease control with low morbidity, it is a therapeutic option that should be taken into account if the patient is not qualified or does not want to undergo surgery. It should also be considered in more advanced stage tumors in which resection would likely to be incomplete or would result in irreversible consequences for the patient.

The exposure of all treatment vantages and disadvantages and likely complications of several treatment methods of pathology is in our opinion the correct approach to allow us to provide better quality medical practice.
